# Osteosarcoma and Metastasis

**DOI:** 10.3389/fonc.2021.780264

**Published:** 2021-12-10

**Authors:** Gaohong Sheng, Yuan Gao, Yong Yang, Hua Wu

**Affiliations:** ^1^ Department of Orthopedics, Tongji Hospital, Tongji Medical College, Huazhong University of Science and Technology, Wuhan, China; ^2^ Department of Oncology, Tongji Hospital, Tongji Medical College, Huazhong University of Science and Technology, Wuhan, China

**Keywords:** osteosarcoma, metastasis, microenvironment, metabolism, noncoding RNAs

## Abstract

Osteosarcoma is the most common primary bone malignancy in adolescents. Its high propensity to metastasize is the leading cause for treatment failure and poor prognosis. Although the research of osteosarcoma has greatly expanded in the past decades, the knowledge and new therapy strategies targeting metastatic progression remain sparse. The prognosis of patients with metastasis is still unsatisfactory. There is resonating urgency for a thorough and deeper understanding of molecular mechanisms underlying osteosarcoma to develop innovative therapies targeting metastasis. Toward the goal of elaborating the characteristics and biological behavior of metastatic osteosarcoma, it is essential to combine the diverse investigations that are performed at molecular, cellular, and animal levels from basic research to clinical translation spanning chemical, physical sciences, and biology. This review focuses on the metastatic process, regulatory networks involving key molecules and signaling pathways, the role of microenvironment, osteoclast, angiogenesis, metabolism, immunity, and noncoding RNAs in osteosarcoma metastasis. The aim of this review is to provide an overview of current research advances, with the hope to discovery druggable targets and promising therapy strategies for osteosarcoma metastasis and thus to overcome this clinical impasse.

## Introduction

Osteosarcoma (OS) is the most common primary malignant bone tumor in children and adolescents and characterized by mesenchymal cells or osteogenic progenitor cells producing osteoid and immature bone (1). The estimated incidence rate of OS is 2–4/million/year worldwide, with first peak at age of 15–19 years (incidence: 8–11/million/year) and second minor peak at age of >60 years (2, 3). OS most commonly occurs in the metaphysis of long extremity bone, such as distal femur, proximal tibia, proximal femur, and proximal humerus, while it rarely arise in axial skeleton and other sites. The 5-year survival rate has reached a plateau in OS patients with localized disease ranging from 60%–70% since the introduction of systematic chemotherapy (4). However, the survival rate of around 20% is still dismal in patients with metastasis (5–7). More importantly, almost all patients are presumed to have subclinical micrometastatic lesions at diagnosis, whereas only 15%–20% of newly diagnosed OS are successfully detected with metastasis (8–10).

The OS cells show a high propensity to disseminate to develop metastasis, which appears to be the most important intrinsic factor for poor outcome of OS patients (5). OS can virtually metastasize to any sites or organs, mostly to lungs and occasionally to bone or lymph nodes (6, 11). The metastatic OS cells from the primary tumor undergo a series of critical steps to colonize and grow in the second site and finally progress into clinically detectable lesions. The biological behavior of metastasis is quite different from primary tumor with respect to cell cycle, differentiation, karyotype, metabolism and surrounding microenvironment, which is caused by differentially expressed genes, shift of molecule profiles, and interaction with microenvironment (12, 13). In the last few decades, a lot of research has been carried out to uncover potential mechanisms underlying OS metastasis and has made encouraging progress. Under the unremitting efforts, more and more biomarkers that are involved in metastasis and prognosis have been discovered. Functional experiments in cells and animal models further validate some remarkable genes and signaling pathways as well as their regulatory patterns responsible for metastasis (14–17). Based on these basic and preclinical studies, many clinical trials have also been initiated to identify novel therapeutic strategies against metastasis. Early diagnosis of metastasis especially micrometastasis will significantly innovate therapeutic modality and doubtlessly improve the prognosis of patients.

In this review, we performed a thorough literature search on OS metastasis and mainly discussed those studies that have been validated *in vivo*. Our focus was primarily on the biological behavior and underlying mechanisms of metastasis. We illustrated the panorama of OS metastasis from various perspectives, such as microenvironment, osteoclast, angiogenesis, metabolism, and immunity. Also, the role of noncoding RNAs in OS metastasis was also discussed, including microRNAs (miRNAs), long noncoding RNAs (lncRNAs), and circular RNAs (circRNAs). The aim of our study is to provide an overall insight into the cross-talk regulatory network in metastasis and to identify core nodes as potential targets for the development of novel therapies. Only breakthroughs in the diagnosis and treatment of metastatic OS can further improve the survival of patients.

## Process of Metastasis

**Figure 1:**
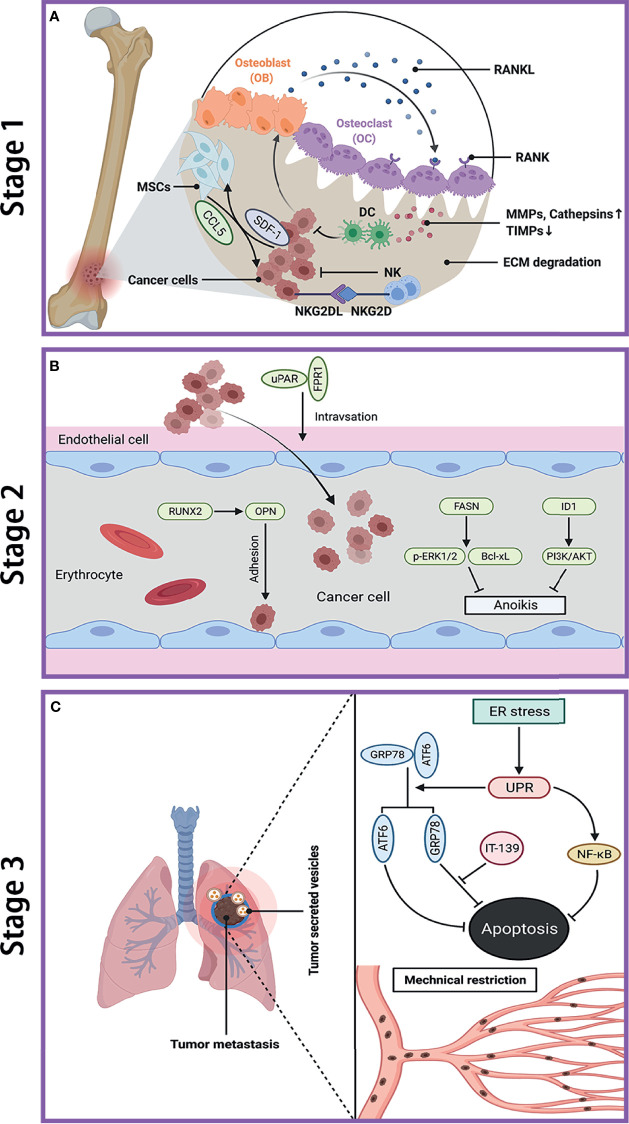
Metastatic cascade of OS to the lung. **(A)** Stage 1: dissemination of metastatic OS cells from primary site. Cancer cells induce OB to secrete RNAKL, which binds to OC, and lead to bone resorption. The increase of MMPs and cathepsins while decrease of TIMPs cause ECM degradation. Cancer cells secret SDF-1 to recruit MSCs, which in turn promote tumor growth and metastasis by secreting CCL-5. NK cells kill cancer cell through the interaction between NKG2D receptor and NKG2D ligands (NKG2DL). **(B)** Stage 2: transfer of OS cells in blood. The interaction between uPAR and FPR1 enhances invasion of cancer cell and their entry into blood. RUNX2/OPN axis promotes adhesion of cancer cell to endothelia cell in lung. FASN and ID1 increase anoikis resistance in cancer cell by upregulating p-ERK1/2 and Bcl-xL and by activating PI3K/AKT pathway, respectively. **(C)** Stage 3: colonization of OS cells in lung. The ER stress-initiated UPR protects cancer cell from apoptosis by activating GRP78 and ATF6, as well as through NF-κB pathway. The mechanical restriction of circulating cancer cells within lung microvasculature partly accounts for the propensity of lung metastasis. Tumor-secreted vesicles reach the lung in advance and direct cancer cell to transfer to the lung. OS, osteosarcoma; OB, osteoblast; OC, osteoclast; RANK, receptor activator of NF-κB; RANKL, receptor activator of NF-κB ligand; MMPs, matrix metalloproteinases; TIMPs, tissue inhibitor of metalloproteinases; ECM, extracellular matrix; SDF-1, stromal cell-derived factor-1; MSCs, mesenchymal stem cells; CCL-5, C–C motif chemokine ligand 5; NKG2D, natural killer group 2 member D; uPAR, urokinase-type plasminogen activator; FPR1, formyl peptide receptor type 1; RUNX2, runt‐related transcription factor 2; OPN, osteopontin; FASN, fatty acid synthase; ID1, inhibitor of differentiation or DNA binding; ERK, extracellular signal-regulated kinase; Bcl-xL, B-cell lymphoma-extra large; ER, endoplasmic reticulum; UPR, unfolded protein response; GRP78, glucose-regulated protein 78KD; ATF6, activating transcription factor 6; IT-139, a novel small molecule that inhibit GRP78.

The dissemination of cancer cells from the primary tumor to a secondary site requires a set of multiple steps, and these metastatic cells exhibit completely distinct characteristics from primary tumor. The pulmonary metastasis process can be divided into three stages, including escape of cancer cells from primary tumor, transit within circulation system, and colonization and establishment of metastatic lesions in lung. Although a large number of tumor cells may gain the potential to enter this metastatic cascade, there are only a few cells that can survive to successfully form metastasis due to the limited efficiency in each step of metastatic cascade (18, 19). Schematic diagram of OS metastasis to the lung is shown in **Figure 1**.

### Stage 1: Dissemination From the Primary Tumor

At the first stage of metastasis, OS cells with an invasive phenotype migrate away from primary tumor and then invade into surrounding tissues. Such process of cancer invasion is critically dependent on the destruction and degradation of basement membrane and extracellular matrix (ECM), which is catalyzed by pericellular and extracellular proteases, mainly by the matrix metalloprotease (MMP) family. Remarkably, a series of studies have suggested that many proteins and microRNAs can promote OS metastasis *via* upregulating various MMPs, including MMP-9 (20, 21), MMP-13 (22, 23), membrane-associated MT1-MMP (also called MMP14) (24), and MMP-16 (25). Cathepsin is another proteolytic enzyme to be involved in OS metastasis (26). Furthermore, some inhibitors targeting MMPs have been explored to suppress OS aggressive behavior by blocking this stage, such as tissue inhibitor of metalloproteinases (TIMPs) (27) and Nobiletin (28). In addition, the interactions between tumor cells and microenvironment such as endothelial cells (29) and mesenchymal stem cells (MSCs) (30, 31) may promote tumorigenicity, whereas interactions with primed dendritic cells (32) and natural killer (NK) cells (33) have antitumor effects. For instance, MSCs promote OS metastasis by the interaction of CCL5 from MSCs and SDF-1 from OS cells *via* autocrine/paracrine communication (31). Tumor lysate-pulsed dendritic cells have been studied in combination with other candidate strategies, such as agonist antiglucocorticoid-induced tumor necrosis factor receptor (anti-GITR) antibody (32), anti-cytotoxic T-lymphocyte antigen-4 (anti-CTLA-4) antibody (33), and antitransforming growth factor-β (anti-TGF-β) antibody (34), to further improve OS treatment *via* enhancing antitumor immunity. In addition, NK cells can kill OS cells, including tumor-initiating cells, through the interaction between natural killer group 2 member D (NKG2D) receptor and its ligand (NKG2DL) (35). The significance of osteoclast-mediated bone destruction and resorption has been revealed throughout the OS development and progression (36). Nevertheless, the exact role of osteoclast in OS metastasis remains controversial and subject to further clarification. A more detailed discussion would be presented in the subsequent part of osteoclasts and metastasis in this review. **Figure 1** **A** depicts the processes of OS cell invasion and migration at primary site.

### Stage 2: Transit Within Circulation System

Firstly, OS cells need to intravasate into microvasculature such as blood vasculature by crossing endothelial cells and basement membrane and then travel within blood flow. The circulating tumor cells arrest and eventually extravasate out from blood into the target secondary organ. Several studies have investigated the interactions between tumor cells and endothelia cells and identified a few related molecules, including runt-related transcription factor 2 (RUNX2), osteopontin (OPN), urokinase-type plasminogen activator (uPAR), and formyl peptide receptor type 1 (FPR1), all of which are further shown to facilitate metastasis *in vivo*. In order to survive in blood vessels, OS cells must acquire anoikis resistance property, which is regulated by many genes (37) such as *FASN* and *ID1*. Additionally, tumor cells also encounter with various physical hemodynamic forces (e.g., fluid shear stress) during transit (38). The time and intensity of circulating tumor cells exposure to fluids will affect their fates either survival or apoptosis (39). The development of bone adapts to mechanical load, suggesting that OS cells might be sensitive to their surround physical stimuli (40, 41). Moreover, the changes in physical stimuli could not only affect biological behaviors of OS cells but also influence their response to chemotherapy and radiation therapy (42, 43). The transfer of OS cells within vasculature is shown in **Figure 1** **B**.

### Stage 3: Colonization and Establishment of Metastasis in Lung

Under a poorly defined mechanism, the majority of circulating tumor cells arrive and arrest in lung microvasculature and subsequently extravasate into lung tissues, whereas only a minority of tumor cells can survive and eventually generate detectable metastasis (19, 44). Compared with primary site, the microenvironment at the secondary site presents a lot of differences, including oxygen tension, nutrition supply, and other physiochemical features. Lung is a foreign microenvironment, where tumor cells will undergo many challenges and face various fates, including apoptosis or death (e.g., immune clearance), dormancy, and proliferation into micrometastasis. In terms of micrometastases, they also have several fates either entering into angiogenic dormancy, or regression, or proliferation to form vascularized macrometastatic lesions (45, 46).

A few studies have attempted to elucidate the potential mechanisms by which OS cells conquer the selective pressures to successfully survive and proliferate in lungs. The response of OS cells to different stresses encountered in lungs is diverse. Endoplasmic reticulum (ER) stress can alter environment in ER lumen and disturb protein folding (47). The unfolded protein response (UPR) and UPR-related signaling pathways are also identified to dysregulated in OS (48, 49). Compared with low metastatic counterparts, OS cells with high metastatic potential express a higher level of ER chaperone protein HSPA5, whose inhibition can reduce lung metastasis (50). The ER stress may even partly account for the resistance of chemotherapy in OS (51). Moreover, the mechanism remains poorly understood by which OS cells preferentially metastasize to lungs. Based on current research, the mechanical restriction of circulating tumor cells within lung microvasculature might play a crucial role in lung tropism (52, 53). Another possible explanation of lung tropism is the notion of premetastatic niche (54). Extracellular vesicles, specifically exosomes released from cancer and stromal cells provide a favorable scenario for initiating organ-directed metastasis in several cancers, including melanoma (55), gastric cancer (56), pancreatic cancer (57), and cervical squamous cell carcinoma (58). A few reviews have discussed the role of exosomes in metastatic organotropism (59, 60), among which one publication focused on bone sarcomas (61). Moreover, Hoshino et al. revealed that tumor-derived exosomes could be uptaken by organ-specific cells to prepare the premetastatic niche, which is mediated by different exosomal integrins depending on the metastatic organs (62). Although their results were mainly from breast cancer, they also profiled the proteome of OS-derived exosome. Recently, several research groups have demonstrated that OS-derived exosomes enhance OS lung metastasis and some circulating plasma exosomal biomarkers are detected to be dysregulated in metastatic OS (63–66). Another study further confirmed the preferential seeding of OS-secreted EVs in lung tissue by fluorescence microscopy with lifetime imaging *in vivo* (67). Thus, we speculate that exosomes derived from metastatic OS cells may preferentially retain in lung to create a premetastatic niche by interacting with resident cells, and thus attract OS cells to migrate to lung and support their survival. More studies are needed to investigate the biodistribution of OS-secreted exosomes and whether molecules within exosomes direct OS cells to lung. A brief description of OS lung metastasis is presented in **Figure 1** **C**.

## Molecular Mechanisms and Promising Targets

As mentioned above, each step of metastatic cascade is rate limited and therefore the key nodes in these steps can serve as potential targets for drug design. Some druggable targets have been summarized in other reviews based on which step the drug acts on (13, 68). Considering the clinical scenario that the majority of patients already have micrometastasis, novel therapy focusing on the later step of metastasis may be more effective. Herein, we provide an overview of biology behavior and regulatory networks of metastasis from various perspectives and summarize current research advances and explore potential targets. The data are extracted from articles, where the results have been validated in animal experiments. Based on the relevance and similarity among these data, we list the core genes and involved signaling pathways or events in **Table 1**. These genes serve as nodes to weave a biological regulatory network of OS metastasis through related pathways, which will continue to be extended and improved with further research. Promising therapy targeting such hub genes or pathways may open a new avenue to treat OS metastasis.

**Table 1:** Summary of genes and signaling pathways involved in osteosarcoma metastasis.

Author	Target gene	Pro/anti	Downstream pathways or events	Author	Target gene	Pro/anti	Downstream pathways or events
Ren et al. (69)	*EZR*	Pro	Lactate production, oxygen consumption↑	Han et al. (70)	*VEGF*	Pro	Meta-analysis
Khanna et al. (16)	*EZR*	Pro	MAPK↑	Jia et al. (71)	*VEGF*	Pro	Angiogenesis↑
Wan et al. (72)	*ITGB4*	Pro	Ezrin↑	Gao et al. (73)	*VEGF*	Pro	Angiogenesis↑
Ren et al. (74)	*PRKC*	Pro	Ezrin↑	Broadhead et al. (75)	*PEDF*	Anti	Angiogenesis↓
Lafleur et al. (76)	*FAS*	Anti	–	Ek et al. (77)	*PEDF*	Anti	Angiogenesis↓
Gordon et al. (78)	*FAS/FASLG*	Anti	–	Ek et al. (79)	*PEDF*	Anti	ALP, pro-α1 collagen, osteocalcin↑
Dhupkar et al. (80)	*PDCD1*	Pro	M2 macrophages, STAT-3/ERK1/2↑	Ek et al. (81)	*PEDF*	Anti	VEGF↓
Lussier et al. (82)	*PDCD1/CD274*	Pro	T-Cell immunity↓	Tang et al. (83)	*CDH4*	Pro	c-Jun/JNK↑
Gvozdenovic et al. (84)	*CD44*	Pro	Hyaluronic acid↑	Dass et al. (85)	*JUN*	Pro	Caspase-1/2/8↓
Gvozdenovic et al. (86)	*CD44*	Anti	Merlin↑	Dass et al. (87)	*JUN*	Pro	Chemosensitivity to doxorubicin↓
Xu et al. (88)	*CD47*	Pro	–	Dass et al. (89)	*JUN*	Pro	Caspase-1/2/8↓
Manara et al. (90)	*CD99*	Anti	Caveolin-1↑ c-Src↓	Sabile et al. (91)	*CCN1*	Pro	–
Adhikari et al. (92)	*CD117*	Pro	CXCR4↑	Habel et al. (93)	*CCN1*	Pro	VEGF, FGF2, PECAM, Ang, MMP-2↑ TSP-1, SPARC↓
He et al. (94)	*CD133*, *CD44*	Pro	–	Habel et al. (95)	*CCN1*	Pro	IGF1/IGF1Rβ, EMT↑
Zhang et al. (96)	*CD151*	Pro	GSK-3β/β-catenin/MMP-9↑	Tu et al. (97)	*IL6*	Pro	STAT3, PCNA↑
Kularatne et al. (98)	*CXCR4*	Pro	–	Zhang et al. (99)	*IL6*	Pro	JAK/STAT3, MAPK/ERK1/2↑
Neklyudova et al. (100)	*CXCL12*	Pro	CXCR4↑	Itoh et al. (101)	*TET2*	Pro	IL-6, MEK/ERK1/2/HIF-1α, ICAM-1↑
Dang et al. (102)	*CXCL5/CXCR2*	Pro	–	Wang et al. (103)	*IL17A/IL17RA*	Pro	VEGF, MMP-9, CXCR4, STAT3↑
Du et al. (104)	*IL8*	Pro	CXCR1/AKT↑	Ségaliny et al. (105)	*IL34*	Pro	Angiogenesis, M2 macrophages↑
Brennecke et al. (106)	*CXCR4/CXCL12*	Pro	AKT, ERK↑	Akiyama et al. (107)	*RANK-Fc*	Anti	RANK/RANKL, ERK↓ anoikis↑
Zhang et al. (108)	*VEGF*	Pro	CXCR4↑	Akiyama et al. (109)	*RANK-Fc*	Anti	RANKL, TRAP, osteoclasts↓
Surmenian et al. (110)	*CXCR7/CXCL12*	Pro	–	Lamoureux et al. (111)	*RANK-Fc*	Anti	RANK, osteolysis↓
Nigris et al. (112)	*YY1*	Pro	VEGF/CXCR4↑	Picarda et al. (113)	*TNFSF10*	Anti	Osteolysis↓ caspase-8↑
Gozo et al. (114)	*FOXC2*	Pro	CXCR4↑	Cao et al. (115)	*SP7*	Anti	Osteolysis↓
Brennecke et al. (116)	*CXCR7*	Pro	CXCL12/CXCR4	Lamora et al. (117)	*SMAD7*	Anti	TGF-β/Smad, TβRI, RANKL↓
Perissinotto et al. (118)	*CXCR4/CXCL12*	Pro	MMP-9↑	Munoz et al. (119)	*ACP5*	Anti	Osteoclasts↑
Li et al. (120)	*DNMT1*	Pro	CXCL12/CXCR4, cytotoxic T-cell homing↓	Calleja et al. (54)	*ΔNTP63α*	Pro	miR-527/665/198↓ SMAD4, TβRII (TGFBR2), KSRP (KHSRP)↑
Kimura et al. (97)	*ITGB1*	Pro	–	Gross et al. (121)	*ΔNTP63*	Pro	IL-6, CXCL8↑
Gvozdenovic et al. (122)	*ITG*	Pro	Anoikis↓ Hippo pathway↑	Bid et al. (123)	*ΔNTP63*	Pro	IL-6/8, STAT-3, HIF-1α, VEGF↑
Li et al. (124)	*NFKB*	Pro	β1 integrin↑	Li et al. (125)	*EDNRA*	Pro	MMP-2↑
Pourebrahim et al. (126)	*TP53*	Anti	Ets2, snoRNAs↓	Zhang et al. (127)	*SALL4*	Pro	Wnt/β-catenin↑
Zhang et al. (128)	*TP53*	Anti	ONZIN/CXCL5/MAPK/ERK↓	Yong et al. (129)	*LDOC1*	Anti	Wnt5a↓
Luther et al. (130)	*IGFBP5*	Anti	C-terminal domain	Wang et al. (131)	*MNAT1*	Pro	AKT1↑
Su et al. (132)	*IGFBP5*	Anti	–	Zeng et al. (133)	*ATF4*	Pro	MTA1/HDAC1↑
Wang et al. (134)	*EFEMP1*	Pro	Wnt/β-catenin, EMT↑	Lu et al. (135)	*IRX1*	Pro	CXCL14/NF-κB↑
Zhang et al. (136)	*EFEMP1*	Pro	PI3K/AKT/mTOR, EMT↑	Manara et al. (137)	*ALP*	Anti	MMP-9↓
Zhang et al. (138)	*S100A4*	Pro	MMP-9↑	Li et al. (139)	*BTG2*	Anti	PI3K/AKT↓
Fujiwara et al. (140)	*S100A4*	Pro	–	Chen et al. (141)	*SLC25A22*	Pro	AKT/FAK↑ PTEN↓
Qin et al. (142)	*TRIM2*	Pro	PI3K/PKB↑	Chien et al. (143)	*NAA10*	Pro	MMP-2↑
Chen et al. (144)	*TRIM66*	Pro	TGF-β, EMT↑	Li et al. (145)	*CDKN1B*	Pro	–
Cao et al. (146)	*WSB1*	Pro	Rac1↑ RhoGDI2↓	Munoz et al. (147)	*PLAU*	Pro	uPAR↑
Fukaya et al. (148)	*PI3K/AKT*	Pro	MMP-2/9↑	Shi et al. (149)	*CRYAB*	Pro	ERK, MMP-9↑
Liu et al. (150)	*AREG*	Pro	ICAM-1↑	Lv et al. (151)	*EZH2*	Pro	H3K27me3↑ TSSC3↓
Guo et al. (152)	*TGFBI*	Pro	α2β1 integrin, PI3K/AKT↑	Zhang et al. (128)	*USP22*	Pro	PI3K/AKT, EMT↑
Baglio et al. (153)	*TGFB*	Pro	IL-6, STAT3↑	Ren et al. (154)	*MIG7*	Pro	Vasculogenic mimicry↑
Liu et al. (155)	*BMI1*	Pro	NF-κB, MMP-9↑	Han et al. (156)	*DNMT3A*	Pro	APCDD1↓ Wnt/β-catenin, EMT↑
Naggar et al. (157)	*HACE1*	Anti	RAC1, ROS↓	Yue et al. (158)	*MAPK7*	Pro	Slug/MMP-9↑
Sun et al. (159)	*ACTL6A*	Pro	EMT↑	Contaldo et al. (160)	*IRS1*	Pro	–
Zhao et al. (161)	*SLC16A1*	Pro	NF-κB↑	Levings et al. (162)	*POU5F1*	Pro	–
Wang et al. (163)	*MTDH*	Pro	NF-κB/EFEMP1/MMP-2↑	Arlt et al. (164)	*FSCN1*	Pro	MMP-9↑
Zandueta et al. (165)	*MGP*	Pro	MMPs, TGF-β/Smad2/3↑	Long et al. (166)	*ALDOA*	Pro	MMP-2↑
Xu et al. (167)	*CEP55*	Pro	AKT, CCND1, FN1↑	Ma et al. (168)	*UBD*	Pro	HOXB9↑
Ren et al. (169)	*PHLDA1*	Pro	ERK1/2, JNK, p38↑	Niinaka et al. (170)	*AMF/GPI*	Pro	vimentin, EMT↑ E-cadherin↓
Shintani et al. (303 )	*DCN*	Anti	–	Tsuru et al. (20)	*HEY1*	Pro	MMP–9↑
Yu et al. (171)	*MED19*	Pro	Cyclin D1/B1, Ki67, PCNA↑ caspase-3, PARP↓	Zhang et al. (172)	*P2RX7*	Pro	PI3K/AKT/GSK3β/β-catenin, mTOR/HIF1α/VEGF↑
Hou et al. (173)	*CCN2*	Pro	integrin/FAK/PI3K/AKT/NF-κB, VCAM-1, αvβ3↑	Zhao et al. (174)	*SPARCL1*	Anti	LRP5/6/Wnt/β-catenin, FZDRs, CXCL5, macrophages↑
McManus et al. (175)	*HES4*	Pro	–	Dai et al. (176)	*RANBP9-PHLDA2*	Anti	AKT↓ anoikis↑
Zhang et al. (177)	*NOTCH*	Pro	HES1↑	Brun et al. (178)	*FHL2*	Pro	Wnt/β-catenin↑
Hughes et al. (179)	*NOTCH*	Pro	Hes1↑	Weekes et al. (70)	*Fos/AP-1*	Pro	FGFR1, MAPK, FRS2α↑
Cheng et al. (180)	*CUL1*	Pro	MMP-9↑	Zhang et al. (181)	*SKP2*	Pro	–
Morrow et al. (182)	Metastatic variant enhancer loci	Pro	BET, AP-1, coagulation factor III/tissue factor (F3)↑	Zhang et al. (183)	*SIRT1*	Pro	–
Zhang et al. (184)	*COPS3*	Pro	Raf-1, Beclin1, MEK/ERK, RSK, EMT, LC3-I /II↑	Lv et al. (185)	*PHLDA2*	Anti	Wnt/β-catenin/Snail/TCF-4, CD44, MMP-7, LRP5, EMT↓ GSK-3β↑
Techavichit et al. (186)	*SFRP2*	Pro	–	Zhao et al. (187)	*PHLDA2*	Anti	Src/PI3K/AKT/mTOR↓ ATG5, autophagy↑
Ma et al. (188)	*CLU*	Anti	Chemoresistance↓	Jin et al. (189)	*PRKDC*	Pro	CyclinD1, PCNA, Bcl-2↑ Bax↓
Hou et al. (190)	*TGFA/EGFR*	Pro	PI3K/AKT/NF-kB, ICAM-1↑	Tieken et al. (191)	*F3*	Pro	IL-8, CXCL-1, SNAIL, MMP-2↑
Chang et al. (192)	*SPON1*	Pro	Fak, Src, MMP-9↑	Yu et al. (193)	*MAD2*	Pro	–
Naggar et al. (194)	*YBX1*	Pro	HIF1α↑	Rubin et al. (195)	*WIF1*	Anti	Wnt↓
Hu et al. (196)	*IDH1*	Anti	–	Cantiani et al. (197)	*CAV1*	Anti	c-Src, MET↓
Sévère et al. (198)	CBL	Anti	RTK, EGFR, PDGFRα↓				

Studies using animal models of osteosarcoma metastasis are included.

Pro, the target gene promotes metastasis; anti, the target gene inhibits metastasis; “↑” upregulation; “↓” downregulation.

### Microenvironment and Metastasis

**Figure 2:**
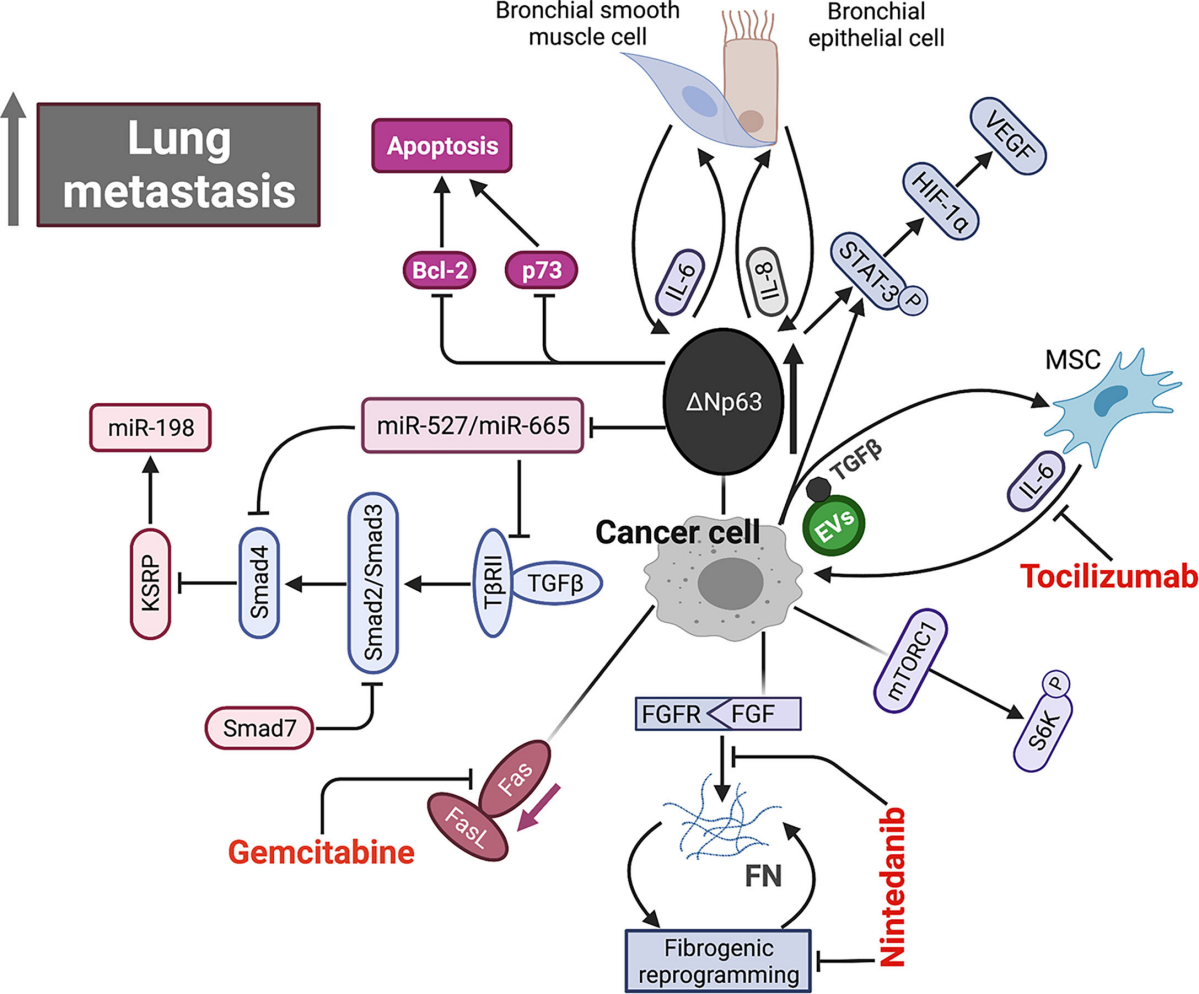
Schematic representation of signaling pathways within microenvironment underlying OS metastasis. Aberrant overexpression of ΔNp63 in cancer cell directly drives feed-forward amplification of IL-6 and IL-8 production by the interactions between cancer cell and both primary bronchial epithelial cell and bronchial smooth muscle cell. ΔNp63 overexpression leads to elevated phosphorylation of STAT-3, which further activates HIF-1α/VEGF axis. High expression of ΔNp63 promotes cancer cell survival by inhibiting Bcl-2 and p73-depedent apoptosis. In addition, ΔNp63 represses miR-527 and miR-665, leading to the upregulation of two TGF-β effectors, Smad4 and TβRII, which inhibits anti-metastasis miR-198 by suppressing its regulatory factor, KSRP. FGF signaling initiates and FN signaling sustains fibrotic reprogramming. Nintedanib targets the pan FGFR-FN axis to inhibit OS lung metastasis. Fas-negative OS cells are selected during metastasis by evading elimination in lung where Fas ligand (FasL) is constitutively expressed. Gemcitabine appears to be a promising agent by upregulating Fas expression. EVs secreted by OS cells selectively incorporate a membrane-associated form of TGF-β and induce IL-6 production by MSCs, which in turn promotes OS progression. IL-6, interleukin-6; IL-8, interleukin-8; STAT-3, signal transducer and activator of transcription 3; HIF-1α, hypoxia-inducible factor 1α; VEGF, vascular endothelial growth factor; TGF-β, tumor growth factor β; FGF, fibroblast growth factor; FN, fibronectin; EVs, extracellular vesicle.

Bone is a type of connective tissue with complex components, including various cells, soluble factors, and extracellular matrix (ECM). These components interact and influence with each other, by which the bone tissue maintains homeostasis in physiological conditions. The appropriate balance between bone formation and destruction determines normal structure of bone. Indeed, OS occurrence and metastatic initiation arise in such a complex bone environment. Additionally, metastatic cells also interact with surrounding microenvironment in each step of metastatic process, primarily in lung. Therefore, study on the interactions between microenvironment and metastatic cells will expand our understanding of OS biology. Increasing evidence has indicated that metastatic cells elicit and receive signals to and from microenvironment, which lead to metastasis inhibition or promotion. **Figure 2** provides a brief overview of the interactions between metastatic OS cells and microenvironment.

*TP63* is a member of the well-known tumor suppressor gene *p53* family, and its splice variant *ΔNTP63* is characterized by the lack of N-terminal transactivation domain. Bid and colleagues have shown that *ΔNTP63* overexpression in OS cells increases phosphorylation of signal transducer and activator of transcription 3 (STAT3) by enhancing interleukin-6 (IL-6) and interleukin-8 (IL-8) secretion (123). In addition, phosphorylation of STAT3 can stabilize hypoxia-inducible factor 1-alpha (HIF1-α) and induce vascular endothelial growth factor (VEGF) secretion. Combined with clinical data, these results reveal a prometastatic role of *ΔNTP63* in OS. Furthermore, the suppression of above cytokine/chemokine signaling pathways can reduce OS metastasis. In a mouse model, the inhibitions of IL-6 and C–X–C motif chemokine 8 (CXCL8, also called IL-8) significantly prolong survival by decreasing their deaths from metastasis (121). Moreover, the combination of inhibitors against IL-6 and CXCL8 achieves an intensive antimetastatic efficiency whereas each inhibitor alone only shows a modest effect. Another laboratory has reported that the metastatic OS cells expressing *ΔNTP63α* disseminate in a transforming growth factor beta (TGFβ)-rich microenvironment by upregulation of Smad4 and TβRII *via* suppressing miRNA-527/665 (120). In addition, *TGF-β* expression on the surface of extracellular vesicles (EVs), derived from OS cells, can induce the IL-6 secretion from mesenchymal stem cells (MSCs), which in turn facilitated proliferation of metastatic cells by activating STAT3. The administration of anti-IL-6 antibody disturbs this cross-talk signaling to reduce metastasis in mice (153). A research group recently has reported that myofibroblastic reprogramming of OS cells contribute to the formation of lung metastasis. This fibrotic reprogramming could be initiated by the activation of fibroblast growth factor (FGF) signaling and sustained by the resultant fibronectin (FN) deposition. They also demonstrated the efficacy of nintedanib in disrupting lung metastasis, but not in primary bone lesion, by blocking fibrotic reprogramming through inhibiting pan FGFR-FN axis (199).

**Figure 3:**
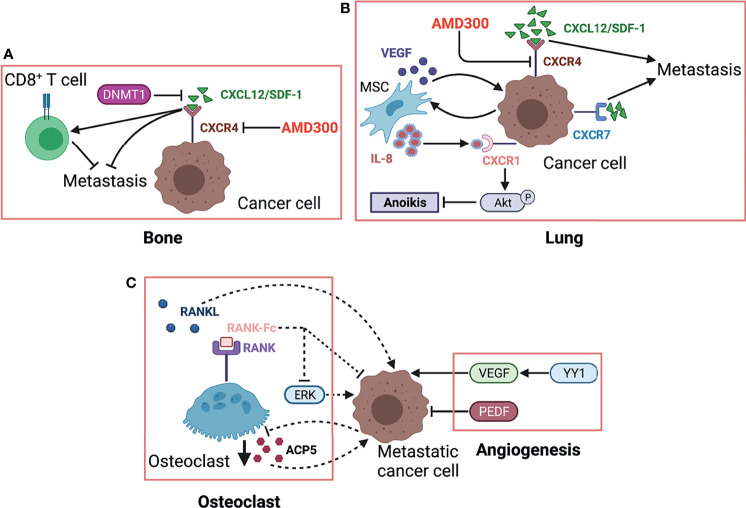
The role of osteoclast and CXCR/CXCL axis in OS metastasis. **(A)** Highly expressed CXCL12. The CXCR4/CXCL12 (SDF-1) interaction is critical for OS metastasis in the lung, which is further strengthened by MSC *via* secreting VEGF. MSC-derived IL-8 induces OS cell anoikis resistance by activating CXCR1/Akt signaling. Another receptor CXCR7 expressed on OS cells promotes lung metastasis and enhances the malignancy activity of CXCR4. **(B)** RANK-Fc binds to RANK as a potent RANKL antagonist to inhibit osteoclast formation and activity, which can reduce OS metastasis, partly by suppressing ERK. Controversially, metastasis-competent OS cells induce loss of ACP5^+^ osteoclasts, which in turn enhances metastasis. Herein, we used dotted lines to indicate this contradiction. VEGF exhibits prometastatic effects on OS cells while PEDF shows the opposite by regulating angiogenesis. **(C)** In primary bone site, OS epigenetically downregulates CXCL12 expression by DNMT1, impairs cytotoxic T-cell homing to the tumor site, and this chemokine gradient of CXCL12 drives the metastasis of OS cells to the lung. ACP5/TRAP, osteoclast-specific tartrate-resistant acid phosphatase 5; PEDF, pigment epithelium-derived factor; YY1, Yin Yang 1 protein; CXCL12, C–X–C motif chemokine 12; DNMT1, DNA methyltransferase 1; CXCR4, chemokine receptor 4; AMD3100, CXCR4 antagonist.

Kleinerman and colleagues have indicated a noteworthy connection between OS cells and resident cells within lung, that is, the metastatic cells expressing Fas will be eliminated by binding to ligand (FasL), which is consistently expressed in lung (76). Under such selective pressure, only those Fas-negative OS cells or cells with nonfunctional Fas signaling can evade this defense mechanism and survive in lung (200). Thus, it is feasible to find agents that can induce Fas expression on OS cells as an alternative therapeutic approach against lung metastasis. This research group has identified two agents, chemotherapeutic agent gemcitabine and histone deacetylase inhibitor entinostat, both of which induce the regression of lung metastasis in wild-type mice by upregulating Fas expression (201, 202). However, such therapeutic efficacy was not observed in FasL-deficient mice since a FasL+ lung microenvironment is a prerequisite for this treatment (78, 203, 204). Moreover, another study confirmed this result again in a canine model with lung metastasis (205). These findings suggest that incorporating lung microenvironment as part of the therapy strategy may benefit patients with established lung metastasis. As an important factor in microenvironment, EVs like tumor-derived exosomes contain various components (e.g., proteins and nucleic acids) and play an essential role in intercellular communication and metastatic progression in a variety of cancers (206). Notably, the expression profiles of EVs from OS samples with low metastatic potential are significantly distinguished from that in high metastatic potential samples by transcriptome analysis (207). A study further indicated that culture medium supplemented with exosomes would change secretome of OS cells and affect their aggressive properties (208). Interestingly, proteomic analysis of the EVs derived from highly metastatic OS cells shows the enrichment of metastasis-related proteins. The EVs from highly metastatic clonal variants of OS can even be internalized into low metastatic cells and thereby endow them with metastatic ability through horizontal phenotypic transfer (67). Also, an *in vivo* metastatic model further confirmed the potential of OS-derived EVs to promote metastasis (65). The exosomes released by OS cells, carrying programmed death-ligand 1 (PD-L1) and N-cadherin, could also stimulate pulmonary metastasis (63), and a recent study found that the plasma exosomal sentrin SUMO-specific protease 1 (SENP1) level is closely related to pulmonary metastasis in OS patients (66). As such, OS cells preferentially migrate and localize to lung directed by the EVs they have secreted, which may partly account for lung tropism of metastasis. The role of cancer-derived exosomes in cancer development and progression was systematically discussed in a review by Kok et al., mainly elaborating on the trafficking of enriched genetic signals carried by exosomal cargo in fostering cancer progression in several tumor types, including OS (209). In addition to exosomes derived from the tumor itself, exosomes from other cells such as bone marrow mesenchymal stem cells (210), adipose-derived mesenchymal stem cells (211) and tumor-associated macrophages can also affect metastasis of osteosarcoma (212, 213). OS cells and non-OS cells in tumor microenvironment could influence themselves or each other by releasing EVs through autocrine/paracrine pathways, shaping tumor microenvironment, modulating cell biological behaviors, especially the aggressiveness of OS cells. The secreted CXCLs within microenvironment selectively recruit different types of cells through binding to their transmembrane receptors. Tumor cells and leukocytes expressing CXCRs migrate following CXCL gradient (214). The CXCL/CXCR axis plays an essential role in leukocyte trafficking, immune homeostasis maintenance like T-cell homing, directional migration of tumor cells. It is well documented that the binding of CXCL12 (also known as SDF-1) to its receptor like CXCR4 or CXCR7 remarkably promotes tumor progression including OS (100, 118, 215). In primary bone site, OS epigenetically downregulates CXCL12 expression by DNMT1, impairs cytotoxic T-cell homing to the tumor site and this chemokine gradient of CXCL12 drives the metastasis of OS cells to the lung (**Figure 3** **A**), where CXCL12 is highly expressed. The constitutive expression of CXCL12 in lung may largely determine lung as the main site of OS metastasis (**Figure 3** **B**). In addition to CXCR4, CXCR7 is another receptor for CXCL12 and also participate in OS lung metastasis (110). The coexpression of CXCR7 and CXCR4 on OS cells could enhance their metastatic ability, since a chemokine gradient of CXCL12 between bone and lung is produced through CXCR7-mediated CXCL12 scavenging in primary bone site (116).Moreover, osteoprotegerin could induce SDF-1 secretion from endothelial cells, which further promotes OS development by increasing neovascularization *via* SDF-1/CXCR4 axis (216). Nigris et al. indicated that transcriptional repressor Yin Yang 1 protein (YY1) enhances metastatic potential of OS cells by activating VEGF/CXCR4 axis (112). Another study also suggested that VEGF secreted from MSCs promotes CXCR4-mediated metastasis (108). In addition, IL-8 produced by MSCs increases anoikis resistance and metastasis of OS cells by regulating CXCR1/Akt signaling pathway (104). Gozo and colleagues has demonstrated that forkhead box protein C2 (FOXC2) maintains OS cells in a stem-like state and promotes metastasis by increasing CXCR4 (114). More importantly, the inhibition of CXCR4 by its antibody (98, 106) or inhibitor (like AMD300) (217) can successfully reduce metastasis. Due to the prominent role of CXCL/CXCR axis in metastasis and its close association with angiogenesis, anoikis, and immune response, novel therapy targeting this axis might be promising for treating metastasis.

### Osteoclasts and Metastasis

The progression of bone tumor leads to osteolysis (218), which in turn promotes the dissemination of tumor cells and thus forms by a vicious cycle. Breaking this vicious cycle between osteoclasts and tumor cells may be one of the promising ways to treat OS metastasis. Several studies have used RANK-Fc to perturb TNFRSF11A/TNFSF11 (also called RANK/RANKL) axis and successfully reduced metastasis by inducing anoikis and apoptosis of OS cells (107, 109, 111). In addition, other agents that can restrict bone resorption are also expected to become potential candidates to inhibit metastasis, such as inhibitors against transcription factor Sp7, TNFSF10 and TGF-β/Smad signaling. Anti-osteoclast drug like zoledronic acid could be synergistically used to enhance the therapy efficacy for OS progression (219). One publication has discussed the prospects of novel therapeutic strategy targeting osteoclast activity for OS (220). However, Munoz et al. suggested the tendency of mutual restrain between metastatic OS cells and osteoclasts and that metastasis-competent OS cells induce the loss of ACP5+ osteoclasts, which in turn facilitate metastasis (119). Notably, EVs derived from OS could suppress osteoclastogenesis and further enhance its metastasis (65). An *in vitro* study was performed to directly explore the reciprocal modulation between OS and osteoclastic cells by a co-culture system (221). More studies are needed to further clarify the exact role of osteoclast in OS metastasis. The interaction between osteoclasts and OS cells was displayed in **Figure 3** **C**.

### Angiogenesis and Metastasis

Angiogenesis is an essential component for tumor growth and progression by supplying adequate blood and nutrients (75, 222). Additionally, these new blood vessels provide the principal route by which cancer cells exit the primary site and enter circulation (223). Angiogenesis is also required for tumor colonization at the site of metastasis. In OS metastasis, intensive investigation has explored the role of angiogenesis and interaction between proangiogenic and antiangiogenic factors, in order to develop potential optimum targets for antiangiogenic therapy (**Figure 3** **A**). A meta-analysis including nine articles revealed that VEGF is positively associated with tumor metastasis and a higher tumor grade (70). Two publications also suggested that angiopoietin-like protein 2 and YY1 accelerate metastasis *via* VEGF-mediated angiogenesis (112, 224). Furthermore, *VEGF* knockdown (73) restricts OS metastasis. Also, the blockade of VEGF receptor 2 (VEGFR2) by anlotinib, a tyrosine kinase inhibitor, results in metastasis suppression in a preclinical study (225). In contrast to VEGF-related angiogenesis, pigment epithelium-derived factor (PEDF) or PEDF-derived synthetic peptides both exhibited antimetastasis activity by inhibiting angiogenesis (77, 79, 81, 226). Antiangiogenesis therapy seems to be an appealing and attractive alternative strategy to manage OS metastasis. However, the safety and efficacy of antiangiogenic therapy has not been confirmed in clinical trials, more work is needed to achieve clinical transformation of this treatment. Current research progress and application limitation in antiangiogenesis therapy for OS can refer to other reviews (227, 228).

### Metabolism and Metastasis

The metabolic program of primary tumor cells is quite different from that of metastatic cells in many ways such as nutritional availability, energy demand, oxygenation level, metabolites, and metabolic pathways, all of which participate in tumor metastasis (229). An advanced metabolomics has shed light on the study of biochemical status and reprogrammed metabolism during metastasis. Metabolomics covers a wide range of metabolites, mainly including sugars, amino acids, and fatty acids. Giang et al. have observed the Warburg effect in several human OS cell lines (230), a common phenomenon of aerobic glycolysis in cancer cells that means pyruvate is transformed into lactic acid even in the presence of oxygen (231). Further study in mice by Hua and colleagues found the dynamic metabolic reprogramming throughout tumor occurrence and progression (232). They identified a number of differentially expressed metabolic biomarkers in serum prior and postmetastasis. Their results suggested the metabolic reprogramming in OS metastasis, characterized by lowered carbohydrate and amino acid metabolism, while an elevated lipid metabolism. Moreover, the serum metabolic profile of lung metastasis is distinct from that of primary tumor. Mice developing lung metastasis have a higher level of lipid metabolites in serum compared with mice without metastasis (232). Consistently, a global analysis of lipidomic reveals the alteration of lipid profiles in metastatic OS cells (233). Previous studies using synvinolin (inhibitor of *de novo* cholesterol synthesis) observed metastasis reduction, which further supported a critical role of lipid metabolism in tumor metastasis (234, 235). The inositol pathway is significantly downregulated in highly metastatic OS cells (236). Functional study both in *ex vivo* lung culture and *in vivo* mouse model has indicated that the administration of inositol hexaphosphate, which will be converted to inositol once entering cells, can reduce lung metastasis by suppressing MAPK and PI3K signaling pathways.

**Figure 4:**
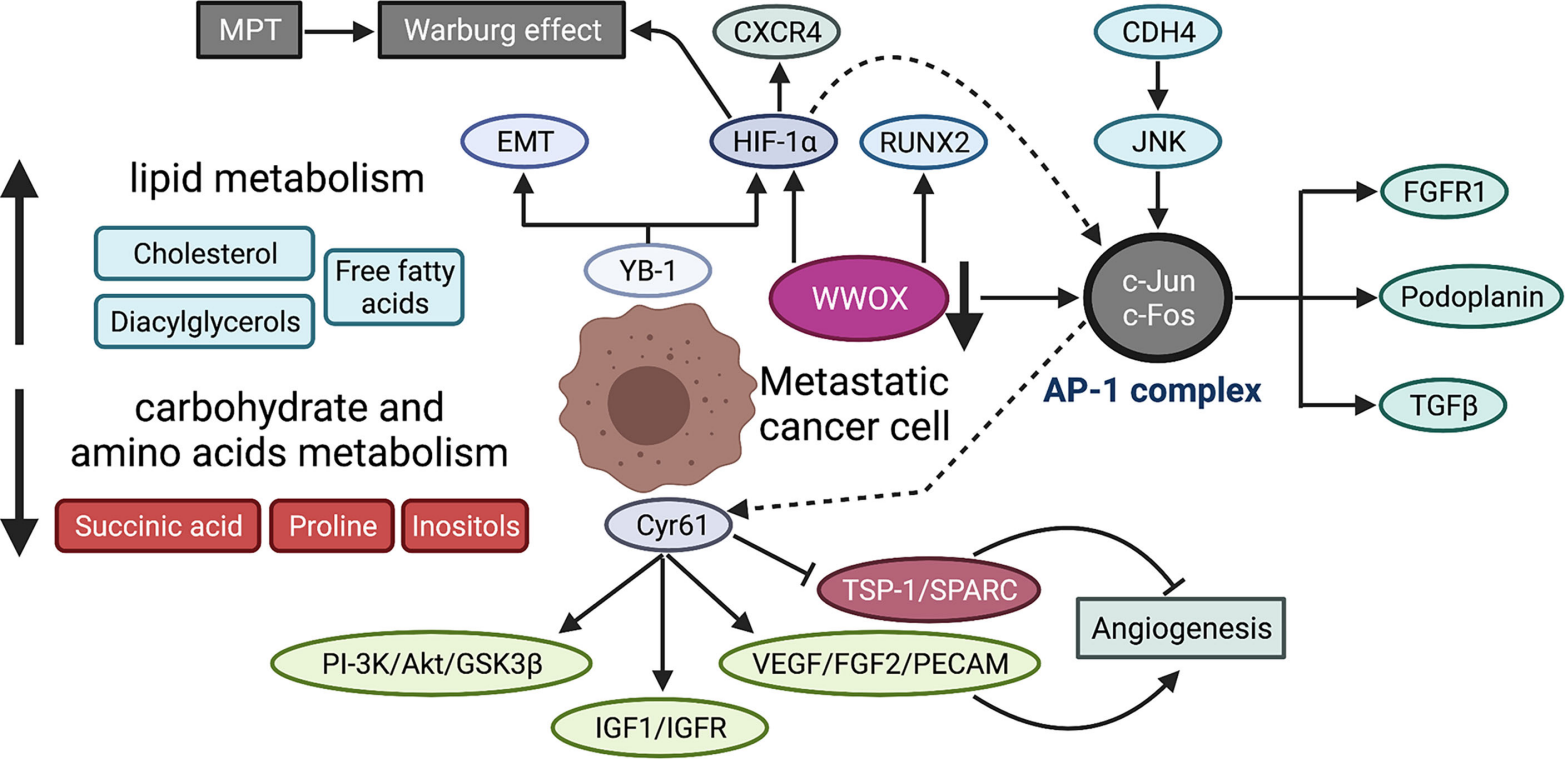
Metabolic reprogramming during OS metastasis. MPT promotes Warburg effect in OS cells by suppressing mitochondrial function. The serum metabolic profile of lung metastasis shows lowered carbohydrate and amino acid metabolism but an elevated lipid metabolism. YB-1 contributes to metastasis by translational activation of EMT and HIF-1α, which then induces CXCR4 expression. Cyr61 enhances the metastatic potential of OS cells through multiple signaling pathways, including PI-3K/Akt/GSK3β, IGF1/IGFR, and angiogenesis-associated signaling (increased VEGF, FGF2, PECAM and reduced TSP-1, SPARC). WWOX maintains mitochondrial respiration and inhibits Warburg effect by physical interaction with HIF-1α. WWOX also suppresses c-Jun activity by physical association while CDH4 overexpression activates c-Jun *via* the JNK pathway. AP-1 is a transcriptional complex, mainly composed of c-Jun and c-Fos, which promotes metastatic potential by upregulating several downstream effectors, including FGFR1, podoplanin, and TGFβ. The dotted lines indicate the mechanistic study is performed in melanoma cells, that is, HIF-1α interacts with AP-1, which then binds to AP-1-binding motif within the Cyr61 promoter and induces Cyr61 expression. MPT, mitochondrial permeability transition; YB-1, Y-box binding protein 1; EMT, epithelial-to-mesenchymal transition; Cyr61, cysteine-rich protein 61; IGF-1, insulin-like growth factor 1; FGF2, fibroblast growth factor 2; PECAM, platelet endothelial cell adhesion molecule; TSP-1, thrombospondin-1; SPARC, secreted protein acidic and rich in cysteine; WWOX, WW domain-containing oxidoreductase; CDH4, cadherin-4; AP-1, activating protein-1; FGFR1, fibroblast growth factor receptor 1.

HIF1-α expression is positively associated with metastasis while negatively with survival. Naggar et al. has shown that Y-box-binding protein 1 (YB-1) facilitates metastasis by direct translational activation of epithelial-to-mesenchymal transition (EMT) and HIF1-α, which then induces CXCR4 expression (194). Functionally, HIF1-α enhances invasion of OS cells in hypoxia *via* increasing VEGF-A (237). Another pathway, HIF1-α/CXCR4 is also proven to facilitate metastasis *in vitro* and *in vivo* (238). Additionally, HIF-1α binds to AP-1-binding motif within Cyr61 (also called CCN1) promoter and induces Cyr61 expression, which plays a prometastatic role in human melanoma cells under hypoxia (239). Hypoxia is one of the prominent features of many malignant tumors and also provides an opportunity to develop agents that target hypoxic region. For instance, hypoxia-activated prodrug TH-302 can reduce OS metastasis as a single agent or in combination with chemotherapy (240). The high expression of Cyr61 indicates poor survival in OS patients (91) and promotes metastasis *via* activating PI-3K/Akt/GSK3β, IGF1/IGFR signaling pathways, facilitating angiogenesis characterized by an increase in VEGF, FGF2, PECAM, and a decrease in TSP-1 and SPARC (93) and promoting EMT-like process (95). Of note, WW domain containing oxidoreductase (*WWOX*) as tumor suppressor has been revealed to maintain mitochondrial respiration and attenuate Warburg effect by inhibiting HIF1-α (241) and c-Jun (242) by physical interaction with them. Recently, a research group further found that WWOX inhibits OS metastasis *in vitro* and *in vivo* through downregulation of RUNX2 (243). The suppression of c-Jun activity can inhibit metastasis by increasing apoptosis (85, 89) and chemosensitivity in OS cells (87). Tang and colleagues demonstrated that cadherin-4 (CDH4) overexpression would activate c-Jun *via* the JNK pathway (83). In addition to c-Jun, proteins from several families like c-Fos, ATF, and MAF, can form transcriptional complex AP-1, and their activation may be one of the mechanisms underlying metastasis (244). Leaner et al. have shown a higher activity of AP-1 in highly metastatic OS cells, compared with low metastatic counterparts (245). In terms of OS, AP-1 promotes metastasis by upregulating podoplanin and TGF-β (246). Another study also reported that FGFR1 silence, a downstream target of c-Fos/AP-1 complex, could significantly reduce lung metastasis (247). The growth and progression of malignant tumors largely rely on glycolysis for energy, resulting in an acidic tumor microenvironment, which may in turn affect the biological behaviors of tumor cells. Several studies have investigated how OS cells adapt to acidic microenvironment. Brief exposure of OS cells to acidic condition results in cell death, whereas prolonged exposure reverses cell death, and even fosters tumor invasiveness (40). The adaption of OS cells to acidosis leads to a metabolic reprogramming with epigenetic stability (248). The underlying mechanism has been further explored, by which acid microenvironment activates a stress-regulated switch to support cell survive. Studies found that cIAP proteins and NF-κB pathway are activated in OS cells in response to acid tumor microenvironment (249, 250). Metabolism-related regulatory networks are shown in **Figure 4**.

As mentioned above, redox stress is a metabolic challenge in lung microenvironment for tumor cells. Studies shown that the excess reactive oxidative species (251, 252) and reactive nitrogen species (253, 254), both impair mitochondrial function. In order to overcome such oxidative pressures, metastatic OS cells trigger their antioxidant response by upregulating redox-related enzymes or glutathione-related metabolic pathways (255, 256). However, the previous mentioned study by Hua et al. reported the contradictory result that glutathione pathway is suppressed at the metastasis phase (232). More studies are urgently needed to further elucidate the exact role of metabolism especially glucose metabolism in tumor cells during dynamic metastatic process. Thereby, the exploration of metabolism reprogramming and metabolic vulnerability may provide novel targets for treating metastasis.

### Immunity and Metastasis

**Figure 5:**
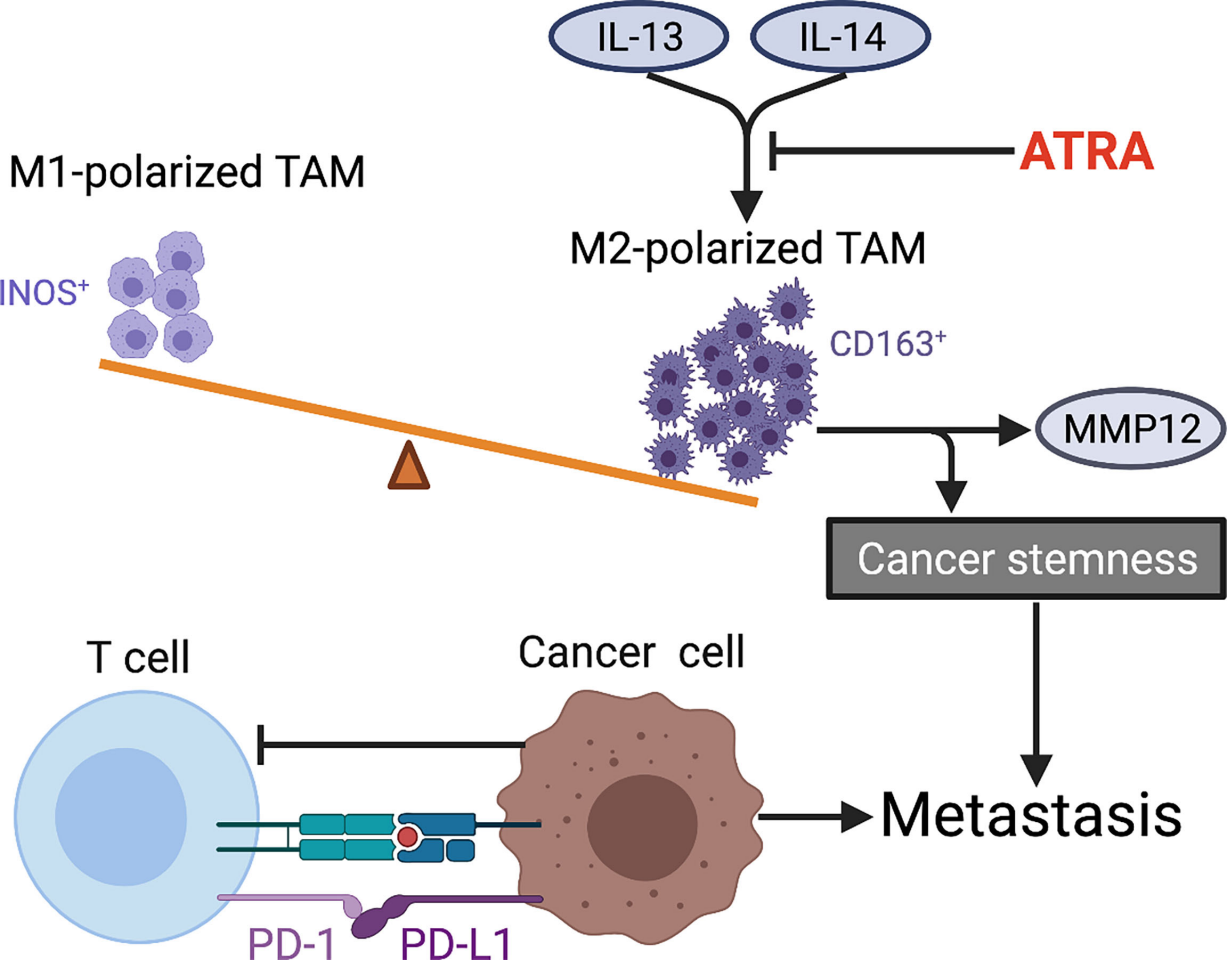
Tumor immune microenvironment characteristics within OS metastasis. The imbalance of M1 (INOS^+^)/M2 (CD163^+^)-polarized TAMs in favor of M2 subtype is observed in metastatic OS. ATRA suppresses IL-13-induced secretion of MMP12 from M2-polarized macrophages and also weakens cancer stemness by preventing M2 polarization of TAMs in OS. The interaction between PD-L1 (expressed in metastatic OS cells) and PD-1 (expressed in tumor-infiltrating CTLs) limits antitumor function of T cells and thus promotes OS metastasis by evading immune surveillance. TAMs, tumor-associated macrophages; ATRA, all-trans retinoic acid; PD-L1, programmed death ligand 1; PD-1, programmed death receptor-1; CTLs, cytotoxic T lymphocytes.

It is well known that immune system both innate and adaptive immunity plays a key role in tumorigenesis and progression, and even to a large extent determines the fate of tumor cells. With the advance in basic and preclinical research, increasing immune-based therapies are being approved for clinical use in various cancers, which yield encouraging outcomes in those patients who are resistant to conventional treatment. Nevertheless, the application of immunotherapy in metastatic OS is far from satisfactory to date. Recently, a series of studies identified several immune cells, including innate immunocytes (e.g., macrophages and dendritic cells) (257–259) and adaptive immunocytes (e.g., T lymphocytes) (260), all of which putatively participate in immune response during OS metastatic progression (**Figure 5**). The number, function, state of immune cells, and their interactions with each other collectively determine whether they promote or inhibit metastasis. Researchers have also recognized some immunocytes as diagnostic or prognostic biomarkers. For instance, the increase of M2-polarized tumor-associated macrophages (TAMs) maintains OS stemness and metastatic property (261). In addition, TAMs activated by mifamurtide (262) or M2-type macrophages suppressed by all-trans retinoic acid (ATRA) (263, 264) both can inhibit tumorigenicity and progression of OS cells. However, the inconsistent results among studies are available, which require more research to further elucidate the dynamic and complex role of TAMs in OS. In addition to TAMs, tumor-infiltrating lymphocytes (TILs) within OS microenvironment also affect OS progression (261, 265). The increased CD8^+^ TILs as well as the high ratio of CD8^+^/FOXP3^+^ TILs both predict a better prognosis in OS patients (266, 267). However, the higher density of TILs is found to be within metastatic sites compared with primary tumor, which constitutes a special immune niche (82, 267). It should be noted that the interaction between PD-L1 (expressed in metastatic OS cells) and PD-1 (expressed in tumor-infiltrating cytotoxic T lymphocytes) limits antitumor function of T cells and thus promotes OS metastasis by evading immune surveillance. The dynamic and intricate regulation of immune system seems to be decisive for metastasis with respect to synthesis of immune factors, spatiotemporal expression of surface markers and phenotypic transition of immunocytes, and their interactions with surrounding compositions. With increasing understanding of immunomodulatory mechanism underlying OS metastasis, researchers are constantly exploring the key immune checkpoints to develop potential immunotherapy strategies for OS with the aim of improving prognosis. A recent review has discussed the mechanisms and status of immunotherapy for OS (268).

## Noncoding RNA

Noncoding RNAs are a large group of RNAs characterized by the lack of ability to encode proteins, which can be divided into regulatory and housekeeping noncoding RNAs (269). Here, we focused on the role of the former in OS metastasis, including microRNAs (miRNAs), long noncoding RNAs (lncRNAs), and circular RNAs (circRNAs). We further outlined the biological function of these noncoding RNAs in OS metastasis and the underlying molecular mechanism. **Table 2** summarizes the noncoding RNAs that have been validated *in vivo*.

**Table 2:** Summary of noncoding RNAs involved in osteosarcoma metastasis.

Author	Noncoding RNA	Pro/anti	Related genes or pathways
**microRNA**			
Gao et al. (270)	miR-17	Pro	PTEN↓
Ding et al. (271)	miR-18a	Anti	MED27, Akt↓
Sun et al. (272)	miR-19	Pro	SOCS6↓; JAK2/STAT3↑
Xin et al. (273)	miR-22	Anti	ACLY, lipogenesis↓
He et al. (274)	miR-23a	Anti	RUNX2, CXCL12↓
Chen et al. (275)	miR-25	Anti	SOX4, EMT↓
Lu et al. (276)	miR-26a	Anti	Jagged1/Notch, ALDH, stemness↓
Zhang et al. (277)	miR-30a	Anti	RUNX2↓
Tao et al. (278)	miR-30a-5p	Anti	FOXD1↓
Zhao et al. (279)	miR-34a	Anti	SIRT1, c-MET, CDK6↓
Liu et al. (280)	miR-92a	Anti	Notch1↓
Yu et al. (281)	miR-124	Anti	TGF-β/Akt/GSK-3β/SNAIL-1↓
Liu et al. (282)	miR-125b	Anti	STAT3↓
Bao et al. (283)	miR-134	Anti	–
Li et al. (284)	miR-137	Anti	FXYD6↓
Shi et al. (285)	miR-139-5p	Anti	DNMT1↓
Gu et al. (286)	miR-140	Anti	–
Xiao et al. (287)	miR-140	Anti	HDAC4↓
Wang et al. (288)	miR-144	Anti	ROCK1, ROCK2↓
Li et al. (289)	miR-145	Anti	CDK6↓
Yang et al. (290)	miR-148a	Anti	ROCK1↓
Zhou et al. (291)	miR-154	Anti	Wnt5a↓
Jiang et al. (292)	miR-181a	Pro	PTEN↓
Zhang et al. (293)	miR-186-5p	Anti	FOXK1, EMT↓
Pan et al. (294)	miR−188	Anti	SOX4↓
Pu et al. (295)	miR-193a-3p; miR-193a-5p	Anti	Rab27B, SRR↓
Li et al. (296)	miR-204-5p	Anti	EBF2↓
Jiang et al. (297)	miR-208b	Anti	ROR2↓
Liu et al. (298)	miR-210	Pro	–
Luo et al. (299)	miR-212	Anti	SOX4↓
Xu et al. (300)	miR-214	Pro	LZTS1↓
Sun et al. (301)	miR-217	Anti	–
Jiang et al. (302)	miR-329	Anti	Rab10↓
He et al. (303)	miR-363	Anti	PDZD2, EMT↓
Xu et al. (304)	miR-372-3p	Anti	FXYD6↓
Li et al. (305)	miR-379	Anti	PDK1↓
Zhao et al. (306)	miR-410	Anti	VEGF↓
Yang et al. (307)	miR-425-5p	Anti	MALAT1, TUG1, Wnt/β-catenin↓
Yuan et al. (308)	miR-451	Anti	–
Yuan et al. (309)	miR−494	Anti	CDK6↓
Qi et al. (310)	miR−496	Anti	eIF4E↓
Pang et al. (311)	miR−497	Anti	–
Cai et al. (312)	miR−590−5p	Anti	KLF5↓
Liu et al. (313)	miR−598	Anti	–
Ma et al. (314)	miR−603	Pro	BRCC2↓
Wang et al. (315)	miR−643	Anti	ZEB1↓
Zhang et al. (316)	miR−663a	Anti	ZBTB7A↓; LncRNA GAS5↑
Liu et al. (317)	miR−873	Anti	HOXA9, Wnt/β−catenin↓
Tanushree et al. (318)	miR−874	Anti	CCNE1↓
Zhong et al. (319)	miR−1270	Anti	–
Yuan et al. (320)	miR−1908	Anti	PTEN↓
**Long noncoding RNA**			
Shi et al. (321)	AFAP1-AS1	Pro	RhoC/ROCK1/p38MAPK/Twist1↑
Li et al. (322)	AFAP1-AS1	Pro	miR−4695−5p↓ TCF4−Wnt/β−catenin↑
Lu et al. (323)	CASC2	Anti	–
Zhao et al. (324)	EPIC1	Anti	MEF2D↓
Zhu et al. (325)	FOXF1-AS1	Pro	FOXF1/MMP2/9↑
Sun et al. (326)	FGFR3-AS1	Pro	FGFR3↑
Ren et al. (327)	FOXD2-AS1	Pro	EZH2, p21↓
Ye et al. (328)	GAS5	Anti	miR-221, EMT↓; ARHI↑
Qu et al. (329)	HOXD-AS1	Pro	STAT3, MMP2↑
Wang et al. (330)	HOTAIR	Pro	MMP2/9↑
Gu et al. (331)	LINC00858	Pro	miR-139↓; CDK14↑
Zhang et al. (332)	LINC01116	Pro	miR-520a-3p↓; IL6R, JAK/STAT↑
Han et al. (333)	LUCAT1	Pro	miR-200c↓; ABCB1↑
Chen et al. (334)	MALAT1	Pro	miR-129-5p↓; RET-PI3K/Akt, stemness↑
Dong et al. (335)	Mal. AT1	Pro	p85α, PI3K/Akt, MMP9, PCNA↑
Duan et al. (336)	MALAT1	Pro	miR−34a↓; cyclin D1↑
Li et al. (337)	miR210HG	Pro	miR-503↓; EMT, N-cadherin, vimentin↑
Hu et al. (338)	NEAT1	Pro	miR-34c↓
Ye et al. (339)	NNT-AS1	Pro	–
Zhu et al. (340)	ODRUL	Pro	miR-3182↓; MMP2↑
Wang et al. (341)	SNHG1	Pro	miR-326↓; NOB1
Jiang et al. (342)	SNHG1	Pro	miR-577↓; WNT2B/Wnt/β-catenin, EMT↑
Deng et al. (343)	SNHG7	Pro	miR-34a↓; TGF-β, SMAD4, EMT↑
Yang et al. (344)	TP73-AS1	Pro	miR-142↓; Rac1↑
Wang et al. (345)	TUG1	Pro	miR-153↓
Yang et al. (346)	XIST	Pro	miR-195-5p↓; YAP↑
Zhang et al. (347)	XIST	Anti^a^	miR-21-5p, EMT↓; PDCD4↑
**Circular RNAs**			
Liu et al. (348)	CircFAT1	Pro	miR-375↓; YAP 1↑

Studies using animal models of osteosarcoma metastasis are included.

Pro, the target gene promotes metastasis; anti, the target gene inhibits metastasis; “↑” upregulation; “↓” downregulation.

a The results in this article is contrary to that of the previous one.

### MicroRNA

MiRNAs contain 18–25 bases and most of them fell within 21–23 bases. Typically, miRNAs bind to 3′untranslated region (3′UTR) of mRNAs by complementarily pairing to destabilize mRNAs or block translation and thus reduce their expressions (349–351). A single miRNA can target multiple mRNAs and vice versa, one mRNA can be regulated by various miRNAs. Thereby, miRNAs may indirectly affect various biological processes such as proliferation, differentiation, apoptosis, and angiogenesis. The unique signatures of miRNA expression profile have been studied in OS metastasis as diagnostic and/or prognostic biomarkers. More importantly, an enormous growing body of studies have explored potential mechanisms, by which miRNAs participate in OS metastasis.

A minority of the miRNAs that we retrieved from literature have been demonstrated for their effects on OS metastasis *in vivo*. Also, only few miRNAs show prometastatic activity in OS, while the majority of miRNAs inhibit metastasis. A study reported that miR-195 suppressed metastatic potential by targeting FASN (352). However, other studies found a high level of miR-195 in serum from OS patients (353, 354). Thus, a single miRNA might play a dual role in different stages of OS progression or the seemingly contradictory results are probably caused by small number of participants. Further studies are needed to confirm the exact function of such miRNAs like miR-195. Whether miRNAs promote or inhibit metastasis depends on proteins they target. Intriguingly, miR-27a* is the first passenger miRNA strand and shows prometastatic activity in OS cells, which is encoded by *MIR27* and directly connects with CBFA2T3, the same as miR-27a (355). Furthermore, the injection of OS cells expressing miR-27a to mice can generate metastatic nodules within lung and bone *in vivo* (356, 357). In turn, miRNAs are also regulated by metastasis-associated genes such as *MYC*, *TP53*, and *TGFB1*. The epigenetic modulation affects the miRNA expression as well. One research team found that apurinic/apyrimidinic endodeoxyribonuclease 1 (*APEX1*) participated in multiple biological processes of OS by shifting miRNA expression profiles (358). Some drugs used to treat OS-like epirubicin (359) and diallyltrisulfide (360) can also alter miRNA expression profiles.

Unfortunately, miRNA-mediated therapy still remains the first phase of clinical trial at best despite so much basic and preclinical research. The main issue needed to be solved is how to deliver miRNAs efficiently and precisely to the sites of interest. Current methods generally show the following deficiencies, including low transfection efficiency, rapid degradation, and abnormal accumulation in nonspecific tissues and organs. Using hyaluronic acid-associated liposomes as carrier may accelerate the entry of miRNAs into cells and prevent them from degradation (361). An alternative approach is to apply viral vectors with hairpin molecules that will be processed into mature miRNAs (362).

### Long Noncoding RNA

LncRNAs are a large group of noncoding RNAs with more than 200 nucleotides and emerging evidence demonstrates its crucial role in multiple biological processes, particularly in tumorigenesis (363, 364). Briefly, lncRNAs in nucleus can regulate gene expression both at genetic and epigenetic levels by affecting transcription factors and chromatin-modifying complexes to bind specific gene loci. In cytoplasm, lncRNAs modulate the stability and translation activity of mRNAs either directly or indirectly. The majority of lncRNAs included in our study compete with miRNAs to bind target mRNAs as sponges. Recently, lncRNAs have engaged much attention in the field of tumor aggressiveness, including migration, invasion, and metastasis. Unlike miRNAs, most of lncRNAs involved in OS are positively associated with metastatic property. We outlined those lncRNAs that have been widely studied in OS metastasis and confirmed *in vivo*.

Metastasis-associated lung adenocarcinoma transcript 1 (MALAT1), also named noncoding nuclear-enriched abundant transcript 2 (NEAT2), is located in chromosome 11q13 with a length of 8.7kb (365). The MALAT1 overexpression is closely correlated with advanced clinical stage and distant metastasis in OS patients (366). Furthermore, a meta-analysis confirmed the predictive value of MALAT1 for metastasis and poor prognosis in six different types of cancer, including OS (367). Consistent with this result, another meta-analysis only including OS also showed MALAT1 as a valuable biomarker for prognosis (368). MALAT1 knockdown inhibits OS metastasis *in vitro* and *in vivo* by affecting various downstream pathways, such as downregulation of PI3K/Akt/MMP-9 signaling pathway (334, 335) and upregulation of E-cadherin and several miRNAs (336, 369). Also, high dose of 17β-estradiol inhibits metastatic potential of OS cells by suppressing MALAT1 while low dose presents the opposite effect (370, 371). It is noteworthy that the most widely studied lncRNAs is the large family of small nucleolar RNA host gene (SNHG), of which all members are considered to promote OS progression and metastasis by sponging various miRNAs. SNHG1, for example, activates Nin one binding protein (NOB1) by sponging miRNA-326 and thus promotes migration/invasion in OS cells and metastasis in murine models (341). Consistently, additional research further shows that SNHG1 is positively related to advanced clinical stage, distant metastasis, and poor survival of OS patients. In addition, SNHG1 facilitates cell proliferation, migration, and invasion *via* activating PI3K/AKT and Wnt/β-catenin pathways by sequestrating various miRNAs (341, 342, 372). Besides, SNHG12 promotes metastasis by increasing angiomotin (373), Notch2 (374) and IGF1R (375) through sponging miRNA-195-5p. There are two research groups that independently demonstrated the role of lncRNA DANCR in OS progression and possible underlying mechanisms. As a competitive endogenous lncRNA, DANCR either increases rho-associated protein kinase 1 (ROCK1) through decoying miRNA-335-5p and miRNA-1972 (376), or enhances AXL/PI3K/Akt signaling pathway by sponging miRNA-33a-5p (377), and thereby promotes OS metastasis.

### Circular RNA

CircRNAs are a newly identified class of endogenous noncoding RNAs characterized by closed-loop structures. Unlike typical linear RNA, circRNAs do not contain 5′-3′ polarity or polyadenylated tail and is thus resistant to enzyme degradation (378, 379). Similar to lncRNAs, circRNAs can competitively bind miRNAs as sponges to indirectly regulate gene expression. Currently, increasing evidence indicates that circRNAs have an important role in many biological behaviors and diseases, particularly in tumorigenesis and tumor progression. The dysregulation of some circRNAs has also been identified in OS metastatic process (380). Almost all circRNAs, like lncRNAs, act as sponges of miRNAs to disinhibit specific prometastatic pathways that normally are inhibited by miRNAs in OS. However, it is not always the case. It has been reported that low expression of circ-HIPK3 is observed in OS cell lines, tissues, and plasm and associated with lung metastasis (381). Overexpression of circ-HIPK3 can inhibit proliferation, migration, and invasion in OS cells. These findings indicate that circ-HIPK3 may have great value in clinical practice as a tumor suppressor. Intriguingly, circ-HIPK3 plays the opposite role in tumor occurrence and progression in other tumors, which strongly suggests that whether a single circRNA acts as an oncogene or a tumor suppressor gene depends on tumor types and which miRNA it sponges (382–386). In addition, circRNAs hsa_circ_0002052 and circ-ITCH are also found to be downregulated in OS cell lines and tissues. Functional studies further confirm that their overexpression can suppress OS cell progressiveness by inhibiting Wnt/β-catenin pathway *via* targeting miRNA-1205/APC2 axis (387), and by suppressing PTEN/PI3K/AKT and Sp1 pathways *via* sponging miRNA-22 (388), respectively. So far, the knowledge and research on circRNAs in OS metastasis are much less than that of miRNAs or lncRNAs. Nevertheless, the special annular structure gives circRNAs unique advantages with stronger stability and higher abundance.

In addition to tumor tissues, collecting noncoding RNAs from blood is convenient in clinical practice. Notably, a number of studies have reported that exosomes could carry noncoding RNAs (212, 213) and even transport them into OS cells (210), thus foster OS metastasis. With the deeper understanding of the relationship between noncoding RNAs and clinicalpathological characteristics, noncoding RNAs will help early diagnosis of metastasis, monitoring of treatment, and prediction of prognosis in OS patients. Although there is no therapy targeting noncoding RNAs used for OS yet, further research will make significant progress to develop novel therapeutic strategies and improve outcomes for OS patients with metastasis.

## Discussion

In conclusion, metastasis is the most important factor resulting in treatment failure and the leading cause of death in OS patients. The 5-year survival rate for patients with metastasis remains only about 20% despite the use of aggressive surgery and intensive chemotherapy. Approximately 20% of patients present detectable metastasis at their first visit to hospital. However, almost all patients with localized disease are assumed to have micrometastasis already and nearly half of them will progress to clinical metastasis.

Although there have been great advances in the research of OS metastasis in the past few decades, the underlying mechanism is not yet clearly elucidated. Generally, the formation of new blood vessels by endothelial cells is a prerequisite for both primary and metastatic tumor growth. For OS, publications suggested that OS cell could facilitate endothelial cell proliferation (389) and several reagents have been tested to suppress angiogenesis of endothelial cells by directly acting on OS cells (390, 391). Although seemingly inconsistent with tumor angiogenesis, OS cells induce contact-dependent endothelial apoptosis, which may contribute to tumor invasion across vascular barrier during metastasis (29). Therefore, the role of endothelial cells and the interaction between them and OS cells are complex, perhaps playing a dual role, or changing dynamically with the stage and site of OS metastasis.

Lung is the most common site of OS metastasis; however, the underlying mechanism of this apparent organotropism remains to be elucidated. Recently, research found that secreted extracellular vesicles, specifically exosomes, would prepopulate in a particular organ, making it more suitable for tumor metastasis. Metastatic organotropism is one of the prominent characteristics of OS, and more investigations are necessary to reveal the potential mechanisms. In addition, novel therapy targeting those molecules that direct OS cells to lung may yield encouraging outcomes in OS patients. Also, immunotherapy has occupied an important position in the field of tumor therapy. However, no immunotherapy has made a breakthrough in metastatic osteosarcoma so far. The role of immune system in OS development and progression may differ from other tumors. Efforts should be made to explore the unique immune niche of OS metastasis, supporting performance of clinical transformation. In addition to external environmental and genetic factors, physical stimuli and modulation significantly influence OS metastatic process. The interaction of metastatic OS cells with their microenvironment, where they encounter dynamic mechanical forces, definitely affects OS cell invasiveness and to some extent determines the preferred metastatic site. However, it is not fully understood how OS cells specifically respond and adapt to their mechanical surroundings. Besides, the substrate stiffness and fluid flow shear stress may also modify the response of OS to therapy. Thus, the research on the fluid flow and/or substrate pressure can provide a novel consideration for developing anti-metastasis therapy.

Herein, we summarize the key regulatory molecules, signaling pathways, and dysregulated noncoding RNAs during metastatic process and hope to reveal potential druggable targets. The innovative technologies have ushered OS metastasis research into new era, such as multi-omics, liquid biopsy, tissue engineering, bioluminescent imaging, and so on. The OS research community should create biological banks and online databases to store and share precious specimens and relevant research results. Bioinformatics is an effective approach to reanalyze the raw data and explore the most valuable information. Moreover, there is a complex regulatory network between microenvironment, angiogenesis, osteoclast, metabolism, immunity, drug resistance, and OS metastasis. Based on emerging technologies and methods, it is expected to depict this network and expand our understanding of metastasis biology.

The standard drug development platform and evaluation system are necessary for clinical translation of promising drugs specifically in rare diseases like OS. As such, it is essential to establish various cell lines and biological models including animal models and bioengineering models that will facilitate research on metastasis. The models especially canine model that faithfully recapitulate metastasis development as in humans are prerequisites for clinical trials of candidate drugs. In addition, we should pay more attention to the effect of drugs on micrometastasis since some drugs uniquely reduce subclinical lesions (392–394). The novel drug delivery strategy and routes may allow conventional drugs to achieve unexpected efficiency while decreasing systemic toxicity (395–397).

The early diagnosis of metastasis and detection of circulating OS cells or micrometastasis will be the hotspots of future research and lead to breakthrough in improving survival of metastatic patients. A sensitive immunomagnetic detection assay has been successfully used to detect micrometastases from bone marrow and peripheral blood (398, 399). Molecular imaging with specific molecular probes are also attempted to track OS cells, such as CXCR4-targeted near-infrared fluorescence imaging (400) and ssDNA aptamer LP-16 targeting metastatic OS cells (401), which both allow detection of OS microlesions.

Drug therapy may be the only option to completely eliminate micrometastasis or inoperable lesions. The increasing studies have identified more and more biomarkers that are of predictive value for metastasis and prognosis in OS patients. However, individualized comprehensive molecular profiling of OS patients has not significantly changed the therapeutic prospects of advanced osteosarcoma. Therefore, more work is needed to better classify patients and further propel personalized management for specific patients. In short, only overcoming metastasis can effectively improve the survival of patients with osteosarcoma.

## Author Contributions

The manuscript was drafted by GS. YG created the diagrams. YY was the guarantor of the entire manuscript for designing and supervising the entire study. HW checked and approved it. All authors contributed to the article and approved the submitted version.

## Funding

This study was supported by the National Natural Science Foundation of China (Grant No. 51537004).

## Conflict of Interest

The authors declare that the research was conducted in the absence of any commercial or financial relationships that could be construed as a potential conflict of interest.

## Publisher’s Note

All claims expressed in this article are solely those of the authors and do not necessarily represent those of their affiliated organizations, or those of the publisher, the editors and the reviewers. Any product that may be evaluated in this article, or claim that may be made by its manufacturer, is not guaranteed or endorsed by the publisher.
